# Beyond “Environment → Outcome”: What taking the “Person × Environment” perspective seriously changes in research practice

**DOI:** 10.3389/fpsyg.2026.1979598

**Published:** 2026-10-29

**Authors:** Giuseppina Spano, Andrea Bosco

**Affiliations:** 1 Department of Psychology and Health Science, Pegaso University, Naples, Italy; 2 Department of Educational Sciences, Psychology, Communications, University of Bari, Bari, Italy

**Keywords:** human-environment relationship, psychological measurement, research agenda, theory development, transactional perspective

## 1 Introduction

Environmental psychology was founded on a distinctive theoretical premise: psychological experience, behavior, and wellbeing emerge from continuous transactions between people and their physical and socio-cultural surroundings (Proshansky et al., 1970; Stokols, 1978). The premise has deep roots; in Lewin's (1936) formulation of behavior as a joint function of person and environment, in Barker's (1968) ecological psychology, and in the transactional worldview articulated by Altman and Rogoff (1987), which treats person and setting as mutually constitutive rather than analytically separable. Unlike frameworks that conceptualize environments as external conditions producing measurable effects on individuals, the discipline has traditionally emphasized the reciprocal, contextual, and temporally extended character of human–environment relationships (Bonnes and Secchiaroli, 1995).

We do not offer Person × Environment (P × E) as a new framework. Previous theoretical perspectives already treat person–environment relations as reciprocal, contextual, and dynamic (Altman and Rogoff, 1987; Barker, 1968), and Ruotolo and Aletta (2026) likewise argue that context is constitutive of, rather than a background moderator to, psychological processes. Our contribution is of a different kind. Ruotolo and Aletta's aim is theoretical integration across mechanisms and scales; ours is narrower and complementary: the study-level logic by which the premise is translated into decisions about design, sampling, modeling, and interpretation, including the sampling of environments and the treatment of heterogeneity. These implications are domain-general and can be applied within, rather than instead of, existing domain-specific frameworks.

Crucially, from the transactional perspective, the “person” is conceptualized as the outcome of a multiplicative, rather than additive, interplay between nature and nurture. Both are constitutive of the person: nature refers to the genetic and biological endowment, while nurture encompasses all non-genetic and non-biological influences, including physical, ecological, social, cultural, and economic environments. These environments are therefore not external factors acting upon a separate individual; through development and ongoing interaction, they become embedded in and constitutive of the person. In this sense, the physical-ecological environment, including both built and natural settings, is a specific dimension of nurture, and the one specifically foregrounded by contemporary Environmental Psychology.

For the purposes of this Opinion, we describe as Environment → Personal (psychological) Outcome (E → PO) a recurring analytical tendency: treating environmental characteristics primarily as independent variables expected to produce measurable psychological consequences. We observe it most clearly in research on restorative environments, green space, and environmental health (Section 2). It is not characteristic of the field as a whole (Ratcliffe et al., 2026): other strands, such as work on place attachment and place identity, are explicitly relational.

This perspective has substantially advanced scientific understanding, but where it predominates it may distance research from the foundational Person × Environment (P × E) perspective, in which psychological processes emerge from the dynamic interplay of people, places, and their histories, identities, relationships, and circumstances. The issue, therefore, is not environmental measurement or quantification *per se*, but the reduction of complex environments and person–environment relations to isolated predictors and outcomes.

We argue that important strands of the field face a tension between methodological refinement and the relational complexity that originally defined Environmental Psychology. Rather than choosing between E → PO and P × E, the next phase should integrate quantitative measurement within multilevel and longitudinal designs that situate psychological outcomes within the broader ecological and relational contexts.

Here, the “ × ” denotes a transactional, mutually constitutive relationship between person and environment, rather than a formal statistical interaction term, although the two senses converge in the moderation models discussed in Section 4.

## 2 From environments as stimuli to environments as relational contexts

One of the most productive contributions of the field has been to demonstrate that physical environments shape human functioning. Research on natural settings, urban green space, restorative environments, and environmental quality has shown that specific environmental characteristics are reliably associated with psychological benefits, including reduced physiological stress, improved directed attention performance, and enhanced wellbeing (e.g., Ulrich et al., 1991; Hartig et al., 2014; Bratman et al., 2019; Spano et al., 2023; Ricciardi et al., 2026). Part of this evidence now supports approximate dose–response formulations linking weekly time spent in nature to self-reported health and wellbeing (White et al., 2019).

Environments do not, however, act on individuals uniformly or deterministically. A place is not merely a stimulus that elicits a response; it acquires psychological significance through the encounter between its physical properties and the person who engages with it. Settings offer affordances that are realized only in relation to the capacities, goals, and states of a particular perceiver. Accordingly, between-person heterogeneity in environmental response is not measurement noise but a substantive feature of the phenomenon.

A forest, a park, or a familiar landscape is not inherently restorative. It may be experienced as restorative because it affords reflection, evokes positive autobiographical memory, signifies safety and belonging, or meets a psychological need at a particular moment (Korpela, 1996; Korpela et al., 2001). Conversely, the same environment may be experienced very differently by individuals differing in history, identity, cultural background, or present circumstance. Place meanings are neither given by the physical setting alone nor freely constructed independently of it (Stedman, 2003).

The distinction matters especially in contexts defined by climate change, environmental uncertainty, and the rapid transformation of familiar places. In this case, environments cannot be understood solely through their measurable effects on wellbeing; they are also sites of identity, attachment, memory, conflict, and adaptation (Adger et al., 2013; Tschakert et al., 2017). In this sense, the constructs of place attachment, i.e., the affective bond individuals develop with specific settings (Scannell and Gifford, 2017), and place identity, i.e., the incorporation of place-related meanings into the self-concept (Twigger-Ross and Uzzell, 1996), have become central to understanding how people relate to threatened or changing environments.

The task for environmental psychology is therefore not only to identify which environmental characteristics produce benefits, but to explain how, for whom, and under what conditions environments become meaningful psychological resources.

## 3 The measurement challenge: preserving meaning in quantitative research

The development of reliable and valid instruments is among the discipline's major achievements. Psychometric work has allowed researchers to operationalize complex concepts and to investigate environmental experience with growing rigor. Yet increasingly refined measures also create a conceptual risk: research questions may come to be shaped by what is readily quantified rather than by the structure of the phenomenon under investigation.

Perceived restorativeness is an instructive case. Attention Restoration Theory and subsequent work on restorative settings have substantially advanced understanding of environmentally supported cognitive recovery (Kaplan and Kaplan, 1989; Kaplan, 1995), and the Perceived Restorativeness Scale (Hartig et al., 1997) has been used extensively to assess the restorative qualities attributed to different environments. The issue is not the validity of such instruments: they are necessary building blocks of rigorous environmental research. Rather, they must be situated within a broader transactional framework.

The broader question concerns which part of the person–environment relation is captured when restoration is measured in isolation from the wider fabric of experience. A restorativeness score describes how a respondent evaluates environmental qualities associated with recovery; it does not, by itself, indicate why a place matters to a particular person, how prior experience conditions present perception, or how identity, autobiographical memory, and social relationships participate in the experience of that place. Critical appraisals of the restorative-environments literature have raised parallel concerns about the specification of the underlying mechanism and about the considerable heterogeneity of effects across studies (Ohly et al., 2016; Joye and Dewitte, 2018).

## 4 Beyond isolated variables: recovering complexity in environmental psychology

Moving beyond the accumulation of isolated variables toward an integrated account of person-environment processes is, in our view, a central task for these strands of research. Figure 1 contrasts the Environment → Personal Outcome (E → PO) perspective with a Person × Environment (P × E) relational framework, in which psychological outcomes emerge from dynamic interactions among environmental, personal, and contextual factors over time.

**Figure 1:**
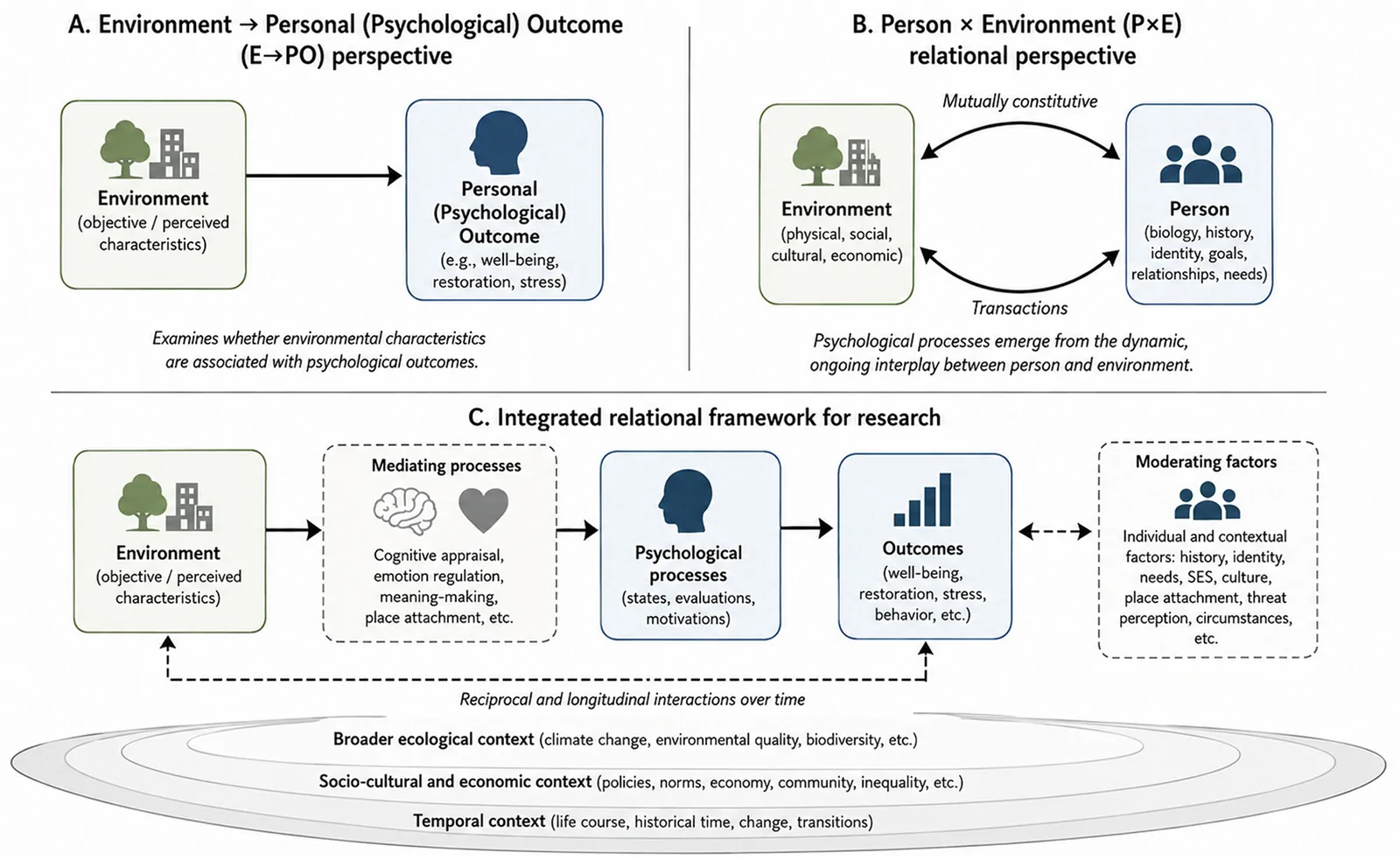
From environmental effects to relational person–environment processes. **(A)** Environment-to-Personal (Psychological) Outcome (E → PO) perspective, examining associations between environmental characteristics and psychological outcomes. **(B)** Person × Environment (P × E) relational perspective, highlighting the mutual constitution of person and environment through ongoing transactions. **(C)** Integrated relational framework for research, incorporating environmental characteristics, mediating psychological processes, outcomes, moderating individual and contextual factors, and broader ecological, socio-cultural, economic, and temporal contexts. Reciprocal and longitudinal interactions are represented by bidirectional and feedback arrows.

A strictly Environment → Personal (Psychological) Outcome approach can establish whether a given environmental feature is associated with a psychological outcome. It is less well equipped to explain why people form intense attachments to threatened places, why communities resist relocation despite documented risk, or why environmental change may generate distress and resilience simultaneously.

Addressing such questions requires acknowledging that environments are not only contexts within which psychological processes occur, but they are constituents of those processes.

This does not imply rejecting quantitative or experimental research but calls for methodological alignment with the relational complexity of the questions being asked.

A few methodological implications follow.

First, understanding how environments affect us requires the explicit modeling of mediating variables. For example, the effect of an objective natural environment on wellbeing may operate through cognitive appraisal and emotional self-regulation processes activated by the individual in a specific context.

Second, understanding for whom and under what conditions environments matter demands the inclusion of moderating variables. Person-level variables, such as biographical history, cultural identity, current needs, pre-existing place attachment, situational circumstances, or perceived climate-related threat, should be specified *a priori* as constituents of the relational hypothesis, rather than introduced *post hoc* as background covariates. Taken together, these implications translate into four concrete research decisions.

In design, person-level moderators and time are specified *a priori* and observed longitudinally or *in situ*, rather than averaged out. In sampling, both people and environments are sampled, with multiple exemplars per environmental category. In modeling, environmental features are not treated as predictors with person variables as covariates; people and environments enter as crossed random effects, with random slopes and moderators or mediators specified as hypotheses. In interpretation, effects are conditional on the sampled persons and places, and variance components are reported and treated as findings rather than as residual error.

A further implication concerns the sampling of environments themselves. Just as adequate sample size and power analysis are used to ensure that findings generalize across individuals, comparable attention is rarely paid to the environments under investigation: hypotheses are often tested using a single exemplar of a given environmental category (e.g., one park, one urban street), and effects are then generalized as though the category itself, rather than the specific setting sampled, had been tested.

Treating environments as a sampled source of variability has a direct statistical consequence. With one exemplar per category, the effect of “green space” cannot be separated from the distinctive features of that place, and uncertainty about generalization to the category is understated (Judd et al., 2012). Only when multiple people encounter multiple environments can person, environment, and person-by-environment variance be estimated separately (Baayen et al., 2008). This reframes heterogeneity: variance in the environment effect across persons or across places is not residual noise but an empirical signature of the relational premise, and explaining it, through the moderators discussed above, becomes a primary aim rather than a robustness check (Bryan et al., 2021).

For instance, a study on the restorative effects of urban green space could sample multiple parks varying in size, vegetation density, and perceived safety, treating these features as continuous, structurally comparable indices rather than as a single undifferentiated “green space” category, and modeling park identity as a crossed random factor alongside participants.

The role of socio-economic status (SES) illustrates how the relational premise changes the modeling and interpretation of a single variable.

Research frequently reports associations between greener neighborhoods and better mental health (e.g., Van den Berg et al., 2015; for an influential review). Income can play different roles in such associations, each corresponding to a different causal model. Under a selection model, higher income increases both the likelihood of living in a green, high-quality area and mental health through other pathways; income is then a confounder of the environment–outcome association, and covariate adjustment is appropriate for estimating the average association net of selection. Under a relational model, income determines which environmental affordances are available and what a given setting means to the person: the same green space may be a basic survival resource for a low-income resident and a lifestyle amenity for someone with greater financial availability. Income is then an effect modifier, and the environment–outcome association differs across income levels.

The two roles are not interchangeable, and they are not mutually exclusive: the same variable can confound the main effect and modify its magnitude, and a model should state which role each term plays. The standard response, including income as a covariate, answers only the first question. It returns a single average effect and assumes that effect is the same at every income level, so income-related heterogeneity is treated as noise to be subtracted rather than as part of the relation under study. Adjustment is also warranted only if income is not itself a consequence of the environment; if environments affected income (e.g., through employment opportunities over time), income would be a mediator, and adjusting for it would remove part of the effect of interest, a form of overcontrol. Which role applies is a matter of causal theory, to be stated explicitly, rather than of statistical convenience.

The relational argument advanced here concerns the second role. Income should not be treated only as a nuisance variable to be neutralized, but also as a structural component of the person–environment relation, which we hypothesize acts as a moderator of environmental effects. We do not hypothesize it as a mediator of the green space–outcome association. Accordingly, models should retain adjustment where a selection process is plausible and add an explicit environment × income term, specified *a priori*, with the interaction interpreted as a substantive finding rather than as a robustness check.

Taken together, the relational premise has consequences at each stage of a study: hypotheses specify for whom and under what conditions an environment matters; samples include both persons and places; models estimate and report how effects vary across them; and interpretations remain conditional on the persons and environments sampled. Taking P × E seriously is therefore less a matter of adding variables than of changing what a study is designed to be able to show.

## 5 Toward a relational future for environmental psychology

Environmental psychology has attained a level of methodological sophistication that has greatly expanded its scientific contribution. The next challenge is to keep that progress tied to the theoretical foundations of the discipline. The distinction between E → PO and P × E should not be read as a choice between competing camps. Nor does E → PO describe the field as a whole: it names a tendency most visible in research on restorative environments, green space, and environmental health. Environmental characteristics do influence psychological experience, and estimating those effects with precision remains essential.

What should be resisted is the reduction of environments to stimuli and psychological experience to measurable outcomes of exposure. The productive path lies in integrating rigorous measurement with renewed attention to relational complexity. The guiding question should not be only what environments do to people, but also what emerges between people and environments and how people make, defend, and remake the places they inhabit.

Ultimately, advancing environmental psychology does not require choosing between scientific rigor and conceptual richness. It requires recognizing that the most rigorous account of human-environment relationships is one that does not lose sight of their inherently relational nature.
